# Prediction of PKCθ Inhibitory Activity Using the Random Forest Algorithm

**DOI:** 10.3390/ijms11093413

**Published:** 2010-09-20

**Authors:** Ming Hao, Yan Li, Yonghua Wang, Shuwei Zhang

**Affiliations:** 1 School of Chemical Engineering, Dalian University of Technology, Dalian, Liaoning 116012, China; E-Mails: dluthm@yeah.net (M.H.); zswei@chem.dlut.edu.cn (S.Z.); 2 Center of Bioinformatics, Northwest A&F University, Yangling, Shaanxi 712100, China; E-Mail: yh_wang@nwsuaf.edu.cn (Y.W.)

**Keywords:** protein kinase C θ, Random Forest, Partial Least Square, Support Vector Machine

## Abstract

This work is devoted to the prediction of a series of 208 structurally diverse PKCθ inhibitors using the Random Forest (RF) based on the Mold^2^ molecular descriptors. The RF model was established and identified as a robust predictor of the experimental pIC_50_ values, producing good external *R*^2^_pred_ of 0.72, a standard error of prediction (*SEP*) of 0.45, for an external prediction set of 51 inhibitors which were not used in the development of QSAR models. By using the RF built-in measure of the relative importance of the descriptors, an important predictor—the number of group donor atoms for H-bonds (with N and O)—has been identified to play a crucial role in PKCθ inhibitory activity. We hope that the developed RF model will be helpful in the screening and prediction of novel unknown PKCθ inhibitory activity.

## 1. Introduction

Playing crucial roles in initiating and controlling immune responses, T cells are responsible for many chronic inflammatory diseases when they are inappropriately or extendedly stimulated [1]. PKCθ is of such importance in the activation and survival of T cells that PKCθ knockout (KO) mice have been reported to have diminished responses in various T cell-mediated disease models, including the experimental autoimmune encephalomyelitis (EAE) model of multiple sclerosis [2,3], the type II collagen-induced arthritis (CIA) model [4], *etc.* Furthermore, these mice exhibited significantly increased survival following a cardiac allograft transplantation, suggesting a potential utility of PKCθ inhibitors as immunosuppressives following transplantation [5]. Interestingly, in spite of the role of PKCθ in immune responses, PKCθ KO mice also have been shown to have normal Th1 differentiation and normal viral clearance despite their defective T cell activation pathway [6]. This suggests that selective PKCθ inhibitors may be able to modulate immunological disorders without imparting severe immunodeficiency. Therefore, PKCθ has become a desirable target for pharmacological intervention of a variety of diseases, especially the T cell-mediated ones [7], such as multiple sclerosis and arthritis. Recently, several classes of compounds, such as pyrimidine analogs and pyridinecarbonitrile derivatives, have been reported as PKCθ inhibitors, illustrating their potential against PKCθ and excellent selectivity over a variety of PKC isoforms [8–17].

Nonetheless, it is well known that the experimental determination for inhibitory activity remains a labor-intensive and time-consuming operation. A more efficient and economical alternative way, *i.e.*, the *in silico* molecular modeling approach, should be employed for the purpose of predicting the endpoints and prioritizing unknown chemicals for subsequent *in vitro* and *in vivo* screening [18]. To the best of our knowledge, however, there is still no report of *in silico* modeling on PKCθ inhibitors. Therefore, it should be beneficial to explore the quantitative structure-activity relationship (QSAR) of structurally diverse PKCθ inhibitors by computational approaches.

Among QSAR investigations, one of the important factors affecting the quality of the model is the molecular descriptors used to extract the structural information, in the form of numerical or digital representation suitable for model development, which serve as the bridge between the molecular structures and physicochemical properties or biological activity of chemicals. A software, Mold^2^ [19], developed by Hong, enables a rapid calculation of a large and diverse set of descriptors encoding two-dimensional chemical structure information. A comparative analysis of Mold^2^ descriptors with those calculated by some typical commercial software packages, such as Cerius^2^ and Dragon, on several data sets using Shannon entropy analysis has demonstrated that Mold^2^ descriptors convey a similar amount of information [19]. Although serving as free available software, Mold^2^ has been proven suitable not only for QSAR analysis, but also for virtual screening of large databases of chemicals due to low computing costs as well as high efficiencies [19].

Another key factor for production of *in silico* models with accurate predictive capabilities, is the selection of appropriate approaches for building the models. Often used statistical methods include; the Multiple Linear Regression (MLR), Partial Least Square (PLS), Linear Discriminant Analysis (LDA), *k*-Nearest Neighbors (*k*NN), Artificial Neural Networks (ANN), Recursive Partitioning (RP), as well as the recent popular, Support Vector Machine (SVM) methods [20]. Though all of them have a proven record of successful applications in QSAR research field, some of them also suffer certain limitations that restrict their generalizations. For example, MLR and LDA—two traditional statistical methods— can only handle data sets where the number of descriptors (*p*) is smaller than the number of molecules (*n*), unless a pre-selection of the descriptors, like by using the genetic or successive projections algorithms, is executed [21,22]; and they are neither flexible nor account for those systems with nonlinear behaviors. ANN and SVM, as relatively new nonlinear techniques in the field of chemometrics employed in classification and regression problems [23], always show low accuracy if too many input variables exist when modeling a dataset. In addition, although these approaches are capable of dealing with high dimensional data, they are not robust to the presence of a large number of irrelevant descriptors, thus requiring the descriptor pre-selection process as well. As a popular computational method, PLS expresses a dependent variable in terms of linear combinations of the independent variables commonly known as principal components. During the modeling process, RP can, on one hand, well handle high dimensional data, while on the other, ignore those irrelevant descriptors, and is free of the aforementioned limitations. However, both the last two methods also have disadvantages: PLS may not be suitable for handling multiple mechanisms of action while RP suffers the drawback that the RP-derived models usually only exhibit relatively low prediction accuracy [18,20,24,25].

Comparatively, the RF [25], based on the ensembles of trees, offers some unique features and does not encounter the limitations stated above. The ensembles of trees are a natural choice of QSAR modeling, since they combine the desirable properties of RP with high prediction performance. Also, RF distinguishes itself from the others due to several merits: (1) RF models are quite resistant to the overfitting problem; (2) RF does not require complicated and time-consuming processes of variable selection; (3) RF can deal with compounds with various mechanisms of actions; and (4) RF performs a faster yet equivalently effective type of cross-validation by using so-called Out-of-Bag (OOB) rather than pure cross-validation which could be computationally cumbersome in some cases. Thus, as a new classification and regression tool, earlier RF has demonstrated to be a very effective solution of QSAR task [20], but which has not yet been widely used in building QSAR models [18,26].

In the present investigation, three popular statistical methods, *i.e.*, the RF, PLS and SVM, were applied and compared on a series of 208 structurally diverse PKCθ inhibitors, with an attempt to build *in silico* models with potent prediction ability. To the best of our knowledge, this is the first attempt to explore the relationship between the molecular structures of PKCθ-related compounds with their PKCθ inhibitory activity. Thus, the aims of this investigation were (1) the development of robust, externally predictive, models based on Mold^2^ descriptors for PKCθ inhibitors; (2) comparison of the performance of the models derived by the three methods of RF, PLS and SVM to determine the superior one (which resulted in the present work as RF); (3) investigation of the influence of tuning parameters on the RF models; and (4) identification of the important descriptors using RF built-in variables’ importance measures.

## 2. Results and Discussion

### 2.1. Performance of RF, PLS and SVM

Currently, random forest, partial least squares and support vector machine—three algorithms popular in chemometrics—were applied on a large dataset of 208 compounds (including 157 molecules as a training set and 51 molecules as a test set) to explore their structure-PKCθ inhibitory activity (expressed by the experimental IC_50_ values). This resulted in one linear model for PLS, and two nonlinear different models for SVM and RF, respectively. All these results were obtained using the R statistical packages, and the pre-processing of the data was performed by the package caret [27]. The statistical performance of the optimum SVM, PLS as well as the RF models using default parameters, is summarized in Table 1.

**Figure 1:**
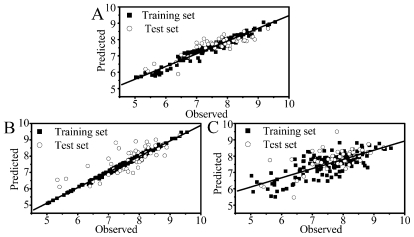
(**A**) Scatter plot of the predicted *versus* observed pIC_50_ values of the RF model; (**B**) scatter plot of the predicted *versus* observed pIC_50_ values of the SVM model; (**C**) scatter plot of the predicted *versus* observed pIC_50_ values of the PLS model.

Using the R package randomForest [28], the RF results were obtained based on the default *m*_try_ (for regression, one-third of the number of descriptors (*p* = 32)) and 500 trees in the forest. For the training set, an *SEE* of 0.25, a coefficient of determination, *R*^2^, of 0.96 are obtained, and for the test set a *SEP* of 0.45 with the coefficient of determination *R*^2^ of 0.76 are obtained, respectively. The *SEP* of the test set is of the same order of magnitude as the *SEE* of the training data, indicating that no overfitting problem exists in the model. In addition, for the OOB process the cross-validated *R*^2^ (*Q*^2^) is 0.54, for the test set the *R*^2^_pred_ is 0.72, suggesting both good internal and external predictions for the developed optimal RF model. Figure 1A shows the performance of the RF model for the training and test sets, respectively, from which it can be clearly concluded that the present RF model exhibits satisfactory predictivity from both the internal and external points of view with respect to the prediction of the test sets.

Support vector machine results were obtained by the R package kernlab [29]. Similar to other multivariate statistical models, the performance of SVM depends on the combination of several parameters, including; the capacity parameter *C*, the kernel type *K* and its corresponding indices. *C* is a regularization parameter which controls the tradeoff between maximizing the margin and minimizing the training error. In this work, the grid search technology was employed to obtain the optimum parameters (*C* and sigma) using the package caret on the basis of 10-fold cross validation. Here, the function sigest in the kernlab package was used to provide a good estimate of the sigma parameter, so that only the *C* parameter was tuned. The final values used in the model were *C* = 100, and sigma = 0.0046 with the lowest root mean square error of 0.59. The SVM model presents a *SEE* of 0.08 for the training and a *SEP* of 0.55 for the test sets, respectively. However, for the training set the *R*^2^ reaches as high as 0.99 with a *Q*^2^ = 0.57, while, for the test one, an *R*^2^ of only 0.61 is obtained with an *R*^2^_pred_ = 0.59, indicating an overfitting problem of the model. Figure 1B illustrates the same tendency where all the train data locate in a straight line, while the test data obviously deviate from the straight line which is a classical overfitting phenomenon.

For the present investigation, could PLS, the widely used linear regression technology in the QSAR model, serving also as the statistical method built-in Comparative Molecular Field Analysis (CoMFA), be applied in building a reliable model? With this question in mind, PLS regression was carried out using the R package PLS [30] for the dataset, with 96 variables resulting from the pre-processing of the original 777 molecular descriptors calculated by the Mold^2^ software solely from the 2D chemical structures. As a result, a 7-latent variable QSAR model was obtained, determined using LOO cross validation with the lowest cross validation root mean square error. The statistical results of the PLS model, present a coefficient of determination *R*^2^ = 0.57, LOO cross validation coefficient *Q*^2^ = 0.36 and *SEE* = 0.59 for the training set, respectively. All these data show that the model is internally bad predictive. The model was also evaluated on unseen chemicals, *i.e.*, the test data, resulted in *R*^2^ = 0.42, *R*^2^_pred_ = 0.39 and *SEP* = 0.67 for the test set, respectively. Figure 1C presents a visual investigation of the PLS scatter plot for predicted *versus* experimental pIC_50_ values of the training and test sets. In a word, PLS generates a relatively poor QSAR model for these PKCθ inhibitors.

### 2.2. Comparison of Different Statistical Approaches

Random Forest, as a new classification and regression tool, has not been frequently applied in QSAR, QSPR (quantitative structure-property relationship), or other chemometrics [26,31–33]. However, Random Forest offers several properties that make it attractive for QSAR analyses, among which the most important ones are: (1) It runs efficiently on high dimensional datasets, and can be used when there are many more variables than observations; (2) it is robust against noise compared to boosting; (3) it does not overfit; (4) it uses a wrapper method that implicitly does feature selection; (5) it can be used both for two-class and multi-class problems and (6) it can handle a mixture of categorical and continuous predictors, *etc*.

Unlike CART (Classification and Regression Trees), each tree in the forest is fully grown without pruning. Due to this, averaging the predictions of many weakly calculated results always ends in a significant performance improvement compared to a single tree. Random forest introduces random training set (bootstrap) and random input vectors into the trees, where each tree is grown using a bootstrap sample of training data at each node, with the best split chosen from a random sample of *m*_try_ variables instead of all variables.

For most QSAR modeling tools, their performance can be significantly influenced if irrelevant descriptors are not removed prior to training. However, descriptors selection optimization, for random forest, is not quite necessary, since the OOB metrics are used in RF to get the estimates of feature importance. Each variable in OOB samples is randomly permuted and the impact of each variable on prediction is measured. Change the value of a parameter *x* and check whether the OOB metrics change dramatically. Variables with high impact are deemed to be important. Meanwhile, the measure of compound’s proximity is also proceeded, which measures how often a pair of points landed in the same terminal node, and this procedure should be useful for outlier detection, clustering, missing value replacement, and low dimensional projections [20]. In the present work, it was demonstrated that RF performed relatively better, although no additional descriptors’ selection was carried out. Also, it can be observed in Table 1, the *R*^2^ and *R*^2^_pred_ for the test set have shown satisfactory statistical predictions according to the general criterion (*R*^2^_pred_ > 0.5, *R*^2^ > 0.6 for the test set) [34].

Gaining popularity recently, SVM has been applied to a wide range of pharmacological and biomedical investigations including drug-likeness [35], drug blood-brain barrier penetration prediction [36], drug receptor binding and drug metabolism [37]. In many cases, SVM was found to be consistently superior to other supervised learning methods when the ratio of samples to variables was at least larger than five [38–40]. However, in this work, SVM model presents an overfitting phenomenon, which might be due to the too low ratio of samples to variables. Whereas, RF still performs well in this investigation, proving its superiority to the SVM for the SAR exploration of PKCθ inhibitors even without pre-processing descriptors.

As a useful regression tool, PLS has been successfully applied in a series of QSAR analyses [18,41]. It applies the latent components to build the final models as a linear technique. In the present work, however, a reliable quantitative structure-activity relationship model cannot be derived by the PLS analysis—this failure might be due to that a linear method may not be suitable for dealing with multiple mechanisms of action. Similar to random forest, there is effectively only one parameter for PLS modeling, that is, the number of the latent components. Different number of components can heavily affect the performance of PLS. Currently, LOO cross validation was used to determine the optimal factors. However, when validated by the independent test set, the PLS model exhibited poor predictive power (*R*^2^ = 0.42 and *R*^2^_pred_ = 0.39, Table 1) compared to the suggested criterion (*R*^2^ > 0.6 and *R*^2^_pred_ > 0.5 for the test set). This implies that these series of PKCθ inhibitors might have multiple mechanisms of action and there may not be proper linear relationship between the inhibition activity of the molecules and their corresponding Mold^2^ indices.

In addition, in order to compare the performance of the RF and PLS models, the *F*-test was applied like in our previous work [41]. Here, the *F*-value is defined as follows:

(1) $$F(n_{1},n_{2})={SEP}_{1}^{2}/{SEP}_{2}^{2}$$

Where *n*_1_ and *n*_2_ are the number of samples in the corresponding test set, and *SEP*_1_^2^ is the square from the higher and *SEP*_2_^2^ is the square from the lower root mean square errors of the two compared models. When comparing the performance of PLS with the RF models, the *F*-value is found to be 2.20, which is higher than the critical one (1.59) indicating a statistically significant difference at a level of significance of 0.95. Since the SVM model has been considered as overfitting, no other comparison was performed presently. To sum up, the statistical test reveals that the performances of RF and PLS technologies are quite different for the series of PKCθ inhibitors. In addition, we also used the *F* test applied to *SEP* and *SEE* to validate the results of the RF method. If the method has predictive value, then the null hypothesis should be supported. That is, *F* = *SEP*^2^/*SEE*^2^ which is 3.24 should be less than the value predicted from the null hypothesis, *ca*. 1.43. 3.24, although close, is greater than to 1.43. The inequality indicates that the model developed from the training set is not sufficient to completely handle the compounds in the test set. The PLS results work out better. In this case, *F* = 1.29 < 1.43, but the *R*^2^ value shows that the PLS method does a much poorer job of developing a model with predictive value. The large value of *F* for the SVM model, 47.27, shows that it has no predictive value. It is well recognized that the development of a QSAR model always involves science and art, and a single method rarely yields perfect and completely unambiguous results, which is just the case here. Our results show that the random forest method is the best of the three algorithms, though the value of *F* indicates that the model, although good, is not complete.

In many QSAR researches [42–45], though only one time split of the training and test sets was carried out, reliable and predictive QSAR models could still be obtained, as long as the criterion that the test set must represent the structural and activity diversity similar to that of the training set was met. However, in many other QSAR studies, for a solid validation of the models, many times of training and test set splits were conducted, as it is well recognized, in terms of statistics, that results from testing one hold-out sometimes is biased for generalization of the models. Thus, in the present work, we also performed 100 times of 51-chemical-hold-out testing (mean and standard deviation) for the RF, SVM, and PLS approaches, with the purpose of achieving a statistically unbiased estimation of the predictive power. Table 2 lists the corresponding results, implying a uniformly worse performance of PLS than that of the RF and SVM. In a word, RF still performs the best in terms of all statistical criteria among the three QSAR methodologies, which conclusion supports again the superiority of RF algorithm than the other two on QSAR study of the large dataset of PKCθ inhibitors.

**Figure 2:**
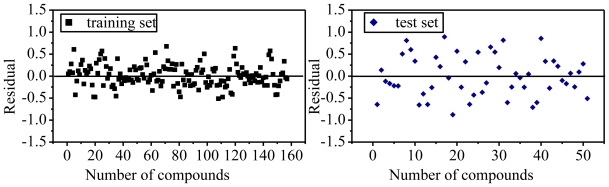
Residual plot for the training and test sets in the RF model.

Outliers from a QSAR are compounds that do not fit the model or are poorly predicted by it [46]. Many reasons may exist for the presence of outliers in the dataset used for *in silico* modeling. Typically, some outliers are recognized as acting by a different mechanism of action from other molecules, which may be well modeled by QSAR techniques, and thus do not follow the general structure-activity rule established by this modeling. When performed correctly, the removal of outliers will allow for the development of stronger and more significant models, and the outlier test is therefore reasonable and necessary in the derived models. There are a variety of methods to highlight outliers including, at the most basic level, identifying those compounds with significantly high residuals from regression-based techniques. At present, since both the SVM and PLS models were considered as failures with the problems of overfitting and poor statistical performance, respectively, only the RF model was checked to identify possible outliers along using the residual plot. As seen from Figure 2, residuals in both the training and test sets distribute evenly above and below the line *y* = 0 and none of them are more than one log unit, illustrating that the RF model is a robust and predictive one. It could be reasonable to consider that there are no outliers in the present RF model.

### 2.3. Focus on the RF Model

From the above results, RF clearly exhibits better statistical performances than the other two models (*i.e.*, PLS and SVM) in this work even without parameters tuning. Thus, our further analysis was only restricted to the RF model for prediction of PKCθ inhibitors.

Most QSAR modeling tools, as we know, require at least a moderate amount of parameter tunings to optimize the performance. Though currently, it has been demonstrated that the RF model performs relatively well “off the shelf”, to investigate the impact of adjusting two key parameters, *i.e.*, *m*_try_ and the number of tree (*ntree*), on the performance of RF, further analyses were performed. Generally, random forest has effectively only one tuning parameter, *m*_try_, which is the number of the descriptors randomly sampled as candidates for splitting at each node during the tree induction. It ranges from 1 to *p*, the total number of descriptors available which case is equivalent to bagging. Choosing *m*_try_ < *p* is usually expected to improve the performance over bagging. However if *m*_try_ = 1, then the trees are essentially making random splits, and the only optimization is the selection of splitting point for the chosen descriptor. One would expect the performance of the ensemble to be suboptimal, unless all descriptors are of equal importance [20]. Generally, *m*_try_ can be chosen to be some function of *p*. The default values of *m*_try_ (*p*/3 for regression) are chosen based on empirical experiments, and the performance of RF seems to change very little over a wide range of values, except near the extremes, *m*_try_ = 1 or *p* [20]. To provide evidence supporting the above claims, 30 replications of 5-fold cross-validation based on the present PKCθ inhibitors data were run, with purpose to assess the correlation between the experimental and test data with a range of *m*_try_ values, including the default ones (32 for regressions).

**Figure 3:**
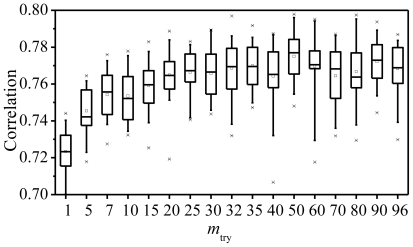
Boxplots of 30 replications of 5-fold cross-validation correlation at various values of *m*_try_ for the PKCθ data set. Horizontal lines inside the boxes are the median correlation.

Figure 3 shows the boxplots of these correlations, with a default value of *m*_try_ of *p*/3 = 32 in this case. This plot suggests that *m*_try_ is optimal when near 50 with a median value of 0.78, while the default *m*_try_ has given a median value of 0.77. It is also observed that the correlation decreases to ~7% when *m*_try_ = 1 compared to the optimum one (*m*_try_ = 50). Therefore, it is still necessary to perform a moderate parameter tuning to get the optimal model, although at most times, RF can give the optimal model by using default parameters, which is supported by a previous report [20].

**Figure 4:**
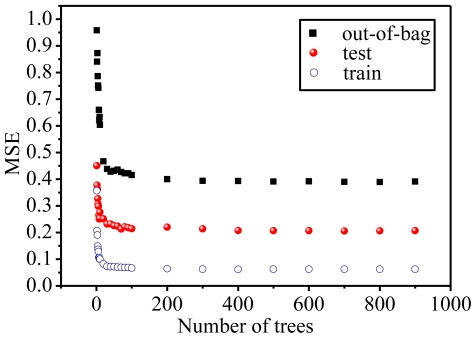
Comparison of the training, out-of-bag, and external test set MSEs for random forest on the PKCθ data set as the number of trees increases.

The other parameter, the number of trees to grow, also affects the performance in most cases: This should not be set to a very small number to ensure that every input row gets predicted at least a few times. In many cases, 500 trees are sufficient (more are needed only if descriptor importance or molecular proximity is desired). There is no penalty for having “too many” trees, other than a waste in computational resources, in contrast to other algorithms which require a stopping rule [20]. To illustrate this, the OOB performance was compared with that of the independent test set for RF as the number of trees increases; Figure 4 shows that a similar tendency exists for the tracks of the OOB MSE and the test set MSE once there are a sufficient number of trees (more than 100 in this work).

Figure 4 also reveals another interesting phenomenon, which is also a typical characteristic of random forest, that is, that the test and OOB MSE do not increase after the training MSE reaches the minimum; instead, they converge to their asymptotic values which are also close to their minimum. In this sense, it can be concluded that RF does not overfit, which has been supported by the similar results of Svetnik [20]. This indicates that RF is a built-in efficient algorithm to evaluate the model prediction ability, where OOB is much faster in the calculation velocity than the cross-validation technology and thus is beneficial to screen those datasets containing a large number of compounds. As seen from Figure 4, when the number of trees approaches 100, the values of MSE in the three sets dramatically converge to their minimum, illustrating that 100 trees are enough to obtain the best performance. Thus, the default 500 trees in the package randomForest are sufficient in this work.

The ideal QSAR model would be robust, sparse, predictive, and interpretable. In many cases such an ideal is not achievable with current descriptors and response variable mapping methods, although much effort is being expended in approaching this ideal. Consequently, QSAR modeling tends to be divided into two classes, depending on the intended outcome of the study. Predictive QSAR aims to screen large, chemically diverse compound libraries that are often noisy, thus they often present less descriptors explanation, especially, with various descriptors like in our work. In addition, in case of possible multiple mechanisms of action among molecules, nonlinear machine learning algorithms are sometimes employed (like SVM, ANN, *etc.*) with purpose of making the corresponding models they built be as potent predictive as possible, so that new candidates can be assessed prior to synthesis or large databases and virtual libraries be screened for hits, which in turn makes the model interpretation much harder. Interpretative modeling often uses linear simulation tools, chemically relevant and interpretable descriptors, and smaller, more congeneric data sets that have usually been measured to a higher degree of accuracy. As a result, it is still a difficult task to produce an as well highly predictive as easily interpretable model. In spite of this fact, we still attempt to offer some rational explanations of the major variables (descriptors) using RF built-in variable importance measure technology.

**Figure 5:**
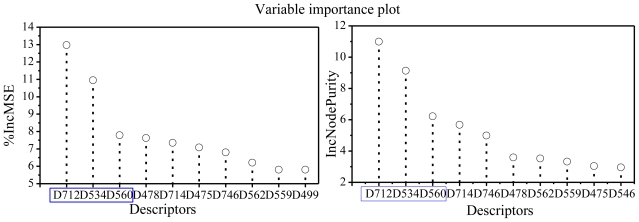
Ordered variable importance scores from RF. The first three important descriptors are surrounded by blue frame.

Here are the definitions of the variable importance measures: (1) Mean Decrease Accuracy (%IncMSE) is constructed by permuting the values of each variable of the test set, recording the prediction and comparing it with the unpermuted test set prediction of the variable (normalized by the standard error). For regression, it is the average increase in squared residuals of the test set when the variable is permuted. A higher %IncMSE value represents a higher variable importance; (2) Mean Decrease Gini (IncNodePurity) measures the quality (NodePurity) of a split for every variable (node) of a tree by means of the Gini index. Every time a split of a node is made on a variable, the gini impurity criterion for the two descendent nodes is less than the parent node. Adding up the gini decreases for each individual variable over all trees in the forest gives a fast variable importance that is often very consistent with the permutation importance measure. As the same as %IncMSE, a higher IncNodePurity value represents a higher variable importance. Figure 5 depicts the variable importance plot. Clearly, three most important variables, *i.e.*, the descriptor D712, D534 and D560, are observed from this figure as measured by the mean decrease in accuracy. Descriptor D712, the number of group donor atoms for H-bonds (with N and O) [19], is found playing the most important role in PKCθ inhibitory activity, which is consistent with experimental results (Table S1). For example, compounds 49, 157, 155 and 156 which have 4, 5, 4 and 4 group donor atoms for H-bonds, respectively, present high inhibitory activity, while compounds 174, 10, 3 and 1 all having only 1 group donor atoms for H-bonds, respectively, exhibit low inhibitory activity. At the same time, one can see that compounds with the values of D712 = 1 are almost inactive ones. Besides, D534 means the lowest eigenvalue from Burdex matrix weighted by masses order-3, and D560 is the lowest eigenvalue from Burdex matrix weighted by polarizabilities order-5. Both of them belong to the Burden eigenvalue descriptors described previously [47]. They are derived from the highest and the lowest eigenvalues of the modified adjacency matrix for the molecules [48]. It is the fact that the Burden eigenvalue descriptors weighted by different properties (atomic masses and polarizabilities) exhibit their importance in the RF model, pointing out that we are dealing with a very complex activity that may depend on different types of molecular interactions. It can be observed from Figure 5 that D712 and D534 present a significantly higher importance than the rest. This indicates that hydrogen bonding interactions play a role in PKCθ inhibitory activity, which is in accordance with the experimental results [10].

In conclusion, though it is often deemed that RF can be used “off the shelf” without expending much effort on parameter tuning or descriptor selection, in some cases it is still important for users to investigate the sensitivity of RF to changes in *m*_try_ which sometimes also influences the performance of the derived QSAR models [18]. By comparison with other statistical methods, RF is found to be a powerful tool producing the top performance exploring the QSAR of PKCθ inhibitors in this work. In addition, the variable importance measure by RF, to some degree, might help researchers find the important variables (descriptors) with corresponding output (biological activity). Therefore, it should be useful for predictive tasks to screen for new and highly potent PKCθ inhibitors.

## 3. Material and Experimental Methods

### 3.1. Data Sets and Descriptors

A large diverse dataset of 220 compounds with the experimental values for the IC_50_ of the PKCθ inhibitors were taken from literatures [8–17], published by the same research group. The activity of all the molecules was measured by the same assay on human PKCθ by employing a modified IMAP protocol from Molecular Devices [8], with IC_50_ values ranging from 0.28 to >30000 nM. Ten chiral compounds with the same 2D structures but different activity were removed due to software limitation. Inhibitors including inequality values reported for two compounds were also deleted for the current QSAR research. Finally, 208 structures with definitive biological values were used for this QSAR analysis. Here, the converted molar pIC_50_ (−log IC_50_) values, ranging from 5.022 to 9.553 nM, were used as the dependent variables in the QSAR regression analysis to improve the normal distribution of the experimental data points. This span of 4 log units of the pIC_50_ values and the unusually large dataset make it highly appropriate for a QSAR analysis.

As for the division rule for training and test sets, various studies have provided different valuable strategies including the most usually used one, *i.e.*, the random selection, and others like the activity-range algorithms, *K*-means-cluster based selection and sphere-exclusion algorithm, *etc*. [43,49–51]. Of the investigations they have made, the separation rule was all emphasized to the key point that how to make the training set to represent the entire data set to the most. In the present work, by random selection 51 compounds were selected as the test set by considering the criterion that the test set must represent both the structural diversity and the range of PKCθ inhibitory activity similar to that of the training set. Table 3 shows several representative compounds together with their activity in the dataset. All information of the 208 compounds with 6 diverse scaffolds of structures used in this work is provided in Table S1 (Supporting Information).

Firstly, the construction of the 2D prediction models depends on the generation of the molecular descriptors. Simply by using various molecular modeling tools, it is possible to calculate thousands of these descriptors directly from the structure of any particular molecule. In this work, a series of 208 two-dimensional structures were drawn with the ISIS/Draw 2.3 program [52], and converted SDF format by open babel software package (http://openbabel.sourceforge.net/). Then the final structures were transferred into Mold^2^, a free program available to public, to calculate the molecular descriptors. Solely from the 2D chemical structures, the Mold^2^ software package can calculate 777 molecular descriptors for each compound, the models generated based on which have been reported comparable to those established based on descriptors calculated by commercial software packages according to Hong *et al.* In our work, firstly each molecule got all original 777 molecular indices by the calculation of Mold^2^ soft. Then, the descriptor preprocessing (often called unsupervised selection of descriptors), which is often required to perform prior to modeling a data set, is executed as follows: (1) Descriptors containing greater than 85% zero values were removed; (2) zero- and near zero- variance predictors were removed because these may cause the model to crash or the fit to be unstable; (3) one of the two descriptors with absolute correlations above 0.75 was omitted; and (4) descriptors with linear combinations were identified and removed correspondingly until the dependencies among predictors were resolved. After these steps, the original 777 descriptors were reduced to 96. The remaining 96 descriptors and their definitions are presented in Supporting Information Tables S2 and S3, respectively.

### 3.2. Statistical Methods

A successful prediction model relies greatly on the use of an appropriate statistical approach. In this work, three popular methods (*i.e*., PLS, SVM and RF) were adopted and compared to develop prediction models for PKCθ inhibitors. Since the detailed theories of the three algorithms have been extensively described in a number of books and literatures [25,53,54], only a brief summary of the methods is provided below.

PLS is similar to principal components regression but with both the independent and dependent variables involved in the generation of the orthogonal latent variables rather than only independent variables used. PLS is based on the projection of the original multivariate data matrices down onto smaller matrices (*T*, *U*) with orthogonal columns, which relates the information in the response matrix *Y* to the systematic variance in the descriptor matrix *X*, as shown below:

(2) $$X=\bar{X}+{TP}^{\prime }+E$$

(3) $$Y=\bar{Y}+{UC}^{\prime }+F$$

(4) U=T+H

Where *X̄* and *Ȳ* are the corresponding mean value matrices, *T* and *U* are the matrices of scores that summarize the *x* and *y* variables respectively, *P* is the matrix of loadings showing the influence of the *x* variables in each component, *C* is the matrix of weights expressing the correlation between *Y* and *T*(*X*) and *E*, *F*, and *H* are the corresponding residuals matrices. PLS calculations also give an auxiliary matrix (PLS weights), which expresses the correlation between *U* and *X* and is used to calculate the *T*. Determination of the significant number of model dimensions was made by cross-validation [55].

Up to date, PLS regression algorithms have been extended to various methods such as the kernel algorithm, the wide kernel algorithm, SIMPLS algorithm and the classical orthogonal scores algorithm. In the present study, the kernel algorithm was selected to build the QSAR models, with leave-one-out (LOO) cross validation used to determine the optimal principal components.

SVM: As a novel type of learning machine, the support vector machine developed by Vapnik and Cortes [54] is based on the structural risk minimization principle from statistical learning theory, and is gaining popularity due to many attractive features and promising empirical performance. Originally, SVM was only developed for solving the protein structural class prediction [56] and other pharmaceutical data analysis problems [57]. With the introduction of *ɛ*-insensitive loss function, up to date SVM has been extended to solve the regression estimation and time series prediction problems with excellent performances obtained [58]. A detailed description of the SVM theory can be referred to several books and papers [54], for which reason the SVM regression is only briefly described here. In support vector regression (SVR), the input is firstly mapped into a higher dimensional feature space by the use of a kernel function, and then a linear model is constructed in this feature space. Any function that meets Mercer’s condition can be used as the kernel function, and the often used kernel functions in the SVM include the linear, polynomial, radial basis function, and sigmoid function, *etc*. For regression tasks, the Gaussian radial basis function kernel is often used due to its effectiveness and speed in the training process. The form of the Gaussian function in R is

(5) $$y=\text{exp}\{-\gamma {\left(\mu -\upsilon \right)}^{2}\}$$

Where; *γ* is the parameter of the kernel, while *μ* and *ν* are two independent variables. The basic idea of SVR is that it approximates the function by minimizing the regularized risk function:

(6) $$R(C)=C\frac{1}{N}\sum_{i=1}^{N}L_{ɛ}(d_{i},y_{i})+\frac{1}{2}{||\omega ||}^{2}$$

Where

(7) $$L_{ɛ}(d,y)=\{\begin{array}{ll} |d-y|-ɛ & |d-y|\geq ɛ\\ 0 & otherwise \end{array}$$

And, *ɛ* is a prescribed parameter. In Equation (6), $C\frac{1}{N}\sum_{i=1}^{N}L_{ɛ}(d_{i},y_{i})$ is the so-called empirical error (risk) measured by the *ɛ*-insensitive loss function *L_ɛ_* (*d*, *y*), which indicates that it does not penalize errors below *ɛ*. The second term, 1/||*ω*||^2^, is used as a measurement of function flatness. *C* is a regularized constant determining the tradeoff between the training error and the model flatness.

RF: RF models were constructed according to the described original RF algorithm [25]. RF is an ensemble of single decision trees, which ensemble produces a corresponding number of outputs and the outputs of all trees are aggregated to obtain one final prediction. The training algorithm of the RF for regression can be briefly summarized as follows: (1) Draw *N* bootstrap samples from the original training set; (2) Construct an unpruned tree *T_p_* (*p* = 1, …, *N*) with each training set *B_p_*. At each node, rather than choosing the best split among all predictors, randomly sample *m*_try_ of the predictors and then choose the best split from among those variables. The tree is grown to maximum size and not pruned back; (3) Predict the *N* trees by average for regression.

RF algorithm is the same as Bagging when *m*_try_ = *p* and the tree growing algorithm used in RF is CART. The RF algorithm can be efficient especially when the number of descriptors (*p*) is very large. This is because RF only tests the *m*_try_ of the descriptors rather than the *p*, where the default *m*_try_ is one-third of the number of descriptors (*p*) for regression. Thus, *m*_try_ is very small, so that the search is very fast. In addition, RF is more efficient than a single tree deriving from that RF does not do any pruning at all, while a single tree needs some pruning using cross validation that can take up a significant portion of the computation time, to get the right model complexity.

RF possesses its own reliable statistical characteristics based on OOB set prediction, which could be used for validation and model selection with no cross-validation performed. It was shown the prediction accuracy of an OOB set and a 5-fold cross validation procedure was near the same [20]. Although RF performs relatively well “off the shelf” without expending much effort on parameter tuning or variable selection [20], it is also of importance for carrying out some tentative investigations on the changes of *m*_try_ or descriptor selection to optimize the performance of RF.

Besides above merits, RF can also calculate descriptor importance in the course of training as follows: As each tree is grown, make predictions on the OOB data for that tree. At the same time each descriptor in the OOB data is randomly permuted on at a time, and each modified data set is also predicted by the tree. Finally, after completing the model training, the margins for each molecule are calculated based on the OOB prediction and OOB prediction with each descriptor permuted. Then the measure of importance for the *j*th descriptor is simply *M* − *M_j_*, where *M* is the average margin based on the OOB prediction and *M_j_* is the average margin based on the OOB prediction with the *j*th descriptor permuted. For regression problems, the margins are replaced by squared prediction errors. The cluster of molecules in descriptor space is usually an important problem in QSAR modeling [59]. However, this is usually done in an unsupervised model like Kennard-Stone [60] using some measures in the descriptor space only. In many applications, researchers might be interested in the proximity measure determined by the subset of those descriptors most relevant to the activity of interest which can be completed by RF. RF predicts a set of molecules whose pairwise proximity is of interest using the model built. The proximity between a pair of molecules is the proportion of trees in the ensemble where the pair landed in the same terminal node. The proximity between a molecule and itself is thus always one. It is apparent that the measure of proximity by RF is supervised because the activity of interest dictates the structure of the tree in the forest, and irrelevant descriptors contribute little to the ensemble, which have little influence on the proximity. Here, we just present a brief introduction about RF, for more details please see the corresponding important literature [20,25].

### 3.3. Evaluation of the Prediction Performance

After the regression model was constructed, the standard error of prediction (*SEP*) and external *R*^2^ (*R*^2^_pred_) [34] parameters for the test set were used to evaluate the model’s predictive performance, which were calculated as follows:

(8) $$SEP=\sqrt{\frac{\sum_{i=1}^{test}{\left(y_{i}-{\hat{y}}_{i}\right)}^{2}}{n}}$$

(9) $$R_{\text{pred}}^{2}=1-\frac{\sum_{i=1}^{test}{\left(y_{i}-{\hat{y}}_{i}\right)}^{2}}{\sum_{i=1}^{test}{\left(y_{i}-{\hat{y}}_{tr}\right)}^{2}}$$

Where *y_i_* and *ŷ_i_* are the measured and predicted (over the test set), respectively, values of the dependent variable, and *ȳ_tr_* is the averaged value of the dependent variable for the training set; *n* is the number of compounds in the test set and the summations run over all compounds in the test set.

## 4. Conclusions

In the present work, a successful computation model was developed, for the first time, for a series of 208 PKCθ inhibitors with diverse scaffolds of structures based on Mold^2^ descriptors using the random forest algorithm. Its statistical results are *R*^2^ = 0.76, *R*^2^_pred_ = 0.72, and *SEP* = 0.45 for the test set, proving potent predictability of the RF model. Among the 96 descriptors used to build the model, three most important ones, *i.e*., D712, D534 and D560, were identified. The analysis of their specification and contribution to the model suggests, on one hand, the crucial role of hydrogen bonding on the interactions between PKCθ and its inhibitors, while on the other, the complex mechanism of action the PKCθ-inhibitor system may involve, which is consistent with the experimental results. Therefore, RF should be a powerful tool to screen highly active PKCθ inhibitors and might also provide some instruction prior to synthesis of a series of new PKCθ inhibitors.

For comparison studies, two alternative approaches—PLS and SVM—were also applied to the dataset. As a result, the problem of overfitting was observed for SVM modeling, while for PLS analysis a poor predictive model with *R*^2^_pred_ = 0.39 and *SEP* = 0.67 for the external dataset was obtained. These results imply, once again, that the PKCθ inhibitors might have multiple mechanisms of action and there may not be a proper linear co-relationship between the inhibitory activity of the molecules and their corresponding Mold^2^ indices. We hope that the adopted model and included above information will be of help for screening and prediction of novel potent PKCθ inhibitors, and for further researches on the subject matter.

## Figures and Tables

**Table 1:** Statistical performance of the QSAR models for PKCθ inhibitors.

Para.^a^	RF		SVM		PLS	

	Training	Test	Training	Test	Training	Test
Size	157	51	157	51	157	51
*R*^2^	0.96	0.76	0.99	0.61	0.57	0.42
*Q*^2^	0.54	-	0.57	-	0.36	-
*R*^2^_pred_	-	0.72	-	0.59	-	0.39
*SEE*	0.25	-	0.08	-	0.59	-
*SEP*	-	0.45	-	0.55	-	0.67

a *R*^2^, coefficient of determination; *Q*^2^, cross-validated *R*^2^: *Q*^2^ based on OOB, 10-fold cross-validation and leave-one-out for RF, SVM and PLS, respectively; *R*^2^_pred_, predictive correlation coefficient for the test set; *SEE*, standard error of estimate; *SEP*, standard error of prediction; -, not applicable or available.

**Table 2:** Statistical performance of QSAR models from 100 times of 51-chemical-hold-out testing (mean and standard deviation) for PKCθ inhibitors.

Para^a^	RF		SVM		PLS	

	Training	Test	Training	Test	Training	Test
Size	157	51	157	51	157	51
*R*^2^	0.95 ± 0.003	0.58 ± 0.09	0.82 ± 0.01	0.49 ± 0.10	0.64 ± 0.13	0.41 ± 0.13
*Q*^2^	0.57 ± 0.03	-	0.59 ± 0.02	-	0.39 ± 0.11	-
*R*^2^_pred_	-	0.56 ± 0.09	-	0.45 ± 0.10	-	0.10 ± 0.84
*SEE*	0.24 ± 0.01	-	0.39 ± 0.01	-	0.53 ± 0.09	-
*SEP*	-	0.59 ± 0.06	-	0.63 ± 0.05	-	0.79 ± 0.25

a *R*^2^, coefficient of determination; *Q*^2^, cross-validated *R*^2^: *Q*^2^ based on OOB, 10-fold cross-validation and leave-one-out for RF, SVM and PLS, respectively; *R*^2^_pred_, predictive correlation coefficient for the test set; *SEE*, standard error of estimate; *SEP*, standard error of prediction; -, not applicable or available.

**Table 3:** Representative chemical structures and inhibitory activity of the PKCθ inhibitor dataset.

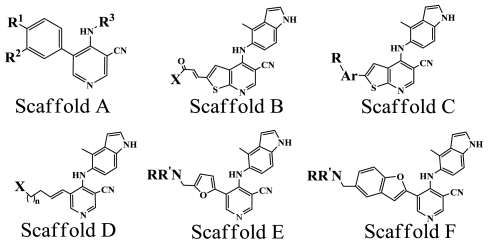						
No.	Scaffold	Substituent			pIC_50_	Ref^a^
		R^1^	R^2^	R^3^		
1*	A	OMe	OMe	3-Bromophenyl	5.337	[8]
2	A	OMe	OMe	Phenyl	5.796	[8]
3*	A	OMe	OMe	3-Chlorophenyl	5.409	[8]
			X			
17	B		Pyrrolidine		8.420	[9]
23	B		H_2_N		7.921	[9]
27	B		PhNH		6.959	[9]
		Ar		R		
77	C	Phenyl		4-CH_2_-NMe_2_	7.854	[11]
80	C	3-Pyridine		5-CH_2_-NMe_2_	7.076	[11]
85*	C	Phenyl		2-OMe,3-CH_2_-NMe_2_	7.921	[11]
		X		*n*		
37	D	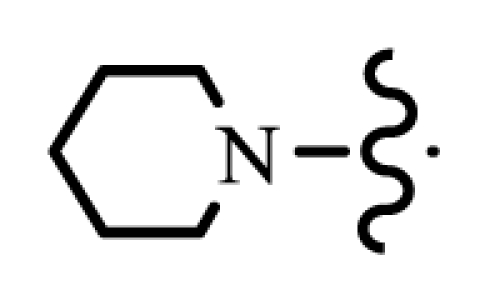		1	7.456	[10]
41*	D	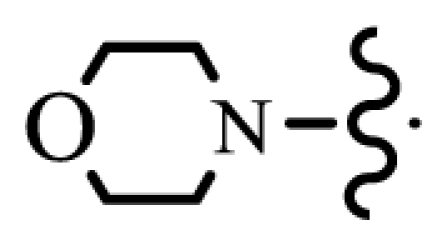		2	7.469	[10]
			NR’R			
137	E		Morpholine		8.108	[14]
140	E		Pyrrolidine		7.456	[14]
			NR’R			
153*	F		Morpholine		7.886	[15]
157*	F		NHCH_2_CH(OH)CH_2_OH		8.824	[15]

* Test set;

a from the corresponding reference.
